# Cost-effectiveness of prucalopride in the treatment of chronic constipation in the Netherlands

**DOI:** 10.3389/fphar.2015.00067

**Published:** 2015-04-14

**Authors:** Mark J. C. Nuijten, Dominique J. Dubois, Alain Joseph, Lieven Annemans

**Affiliations:** ^1^Market Access and Health Economics, Ars Accessus Medica BVJisp, Netherlands; ^2^Pharmed, Université Libre de BruxellesBrussels, Belgium; ^3^Health Economics and Health Outcomes, Shire InternationalNyon, Switzerland; ^4^Department of Public Health, Ghent UniversityGhent, Belgium

**Keywords:** prucalopride, cost-effectiveness, chronic constipation, Markov model, laxatives

## Abstract

**Objective:** To assess the cost-effectiveness of prucalopride vs. continued laxative treatment for chronic constipation in patients in the Netherlands in whom laxatives have failed to provide adequate relief.

**Methods:** A Markov model was developed to estimate the cost-effectiveness of prucalopride in patients with chronic constipation receiving standard laxative treatment from the perspective of Dutch payers in 2011. Data sources included published prucalopride clinical trials, published Dutch price/tariff lists, and national population statistics. The model simulated the clinical and economic outcomes associated with prucalopride vs. standard treatment and had a cycle length of 1 month and a follow-up time of 1 year. Response to treatment was defined as the proportion of patients who achieved “normal bowel function”. One-way and probabilistic sensitivity analyses were conducted to test the robustness of the base case.

**Results:** In the base case analysis, the cost of prucalopride relative to continued laxative treatment was € 9015 per quality-adjusted life-year (QALY). Extensive sensitivity analyses and scenario analyses confirmed that the base case cost-effectiveness estimate was robust. One-way sensitivity analyses showed that the model was most sensitive in response to prucalopride; incremental cost-effectiveness ratios ranged from € 6475 to 15,380 per QALY. Probabilistic sensitivity analyses indicated that there is a greater than 80% probability that prucalopride would be cost-effective compared with continued standard treatment, assuming a willingness-to-pay threshold of € 20,000 per QALY from a Dutch societal perspective. A scenario analysis was performed for women only, which resulted in a cost-effectiveness ratio of € 7773 per QALY.

**Conclusion:** Prucalopride was cost-effective in a Dutch patient population, as well as in a women-only subgroup, who had chronic constipation and who obtained inadequate relief from laxatives.

## Introduction

Constipation is a common condition that may be managed by self-medication or medical consultation. A consensus definition of chronic constipation, known as the Rome III criteria, requires a patient to have experienced at least two of the following symptoms over the previous 3 months: fewer than three bowel movements per week; at least 25% of defecations with straining; lumpy or hard stools; sensation of anorectal obstruction; sensation of incomplete defecation; and manual manipulation required to defecate. In addition, the Rome III criteria note that loose stools are rarely present without the use of laxatives and that a patient's symptoms should not meet the defined criteria for irritable bowel syndrome. Although symptoms of constipation are assessed over the previous 3 months, patients must be symptomatic for at least 6 months prior to diagnosis to fulfill the criteria for chronic constipation (Longstreth et al., 2006).

Most patients with chronic constipation should first attempt lifestyle modifications such as increasing dietary fiber intake to 15–20 g per day, drinking plenty of fluids, and maintaining a regular exercise program. Despite limited data supporting their use in clinical practice, these lifestyle changes promote general health and may improve bowel symptoms in some patients (Chung et al., 1999; Meshkinpour et al., 1998; Annells and Koch, 2003; Dukas et al., 2003). The short-term benefits of laxatives are undeniable. Laxatives have been shown to increase stool output and frequency (Passmore et al., 1993) and are relatively straightforward to administer. However, the management of chronic constipation is multifaceted and complex (Dennison et al., 2005).

Chronic use of laxatives may have serious adverse effects and excessive laxative use has been linked to loss of colonic motility (Read et al., 1995). Other less severe, but more common, adverse effects of laxatives include headache, loss of appetite, and diarrhea (Passmore et al., 1993; Romero et al., 1996). Duncan et al. (1992) reported that 4% of new cases of diarrhea among patients seen in gastroenterology clinics were laxative-induced. All of these consequences may negatively impact on health-related quality of life (HRQoL) and may precipitate frequent physician visits and diagnostic testing (Duncan and Forrest, 2001). Although the study by Duncan et al. was performed in patients with a recent history of constipation, we might expect a similar trend in individuals with chronic constipation.

Chronic constipation is associated with various comorbidities and, although rare, there are several potentially life-threatening complications (Singh et al., 2005). The most common complications are anorectal disorders such as hemorrhoids, fissures, rectal prolapse, and fecal impaction. Severe refractory chronic constipation may eventually require a partial colectomy to be performed (Pikarsky et al., 2001). Such surgical treatment should be considered only in the most severe cases, for those patients who do not respond to aggressive medical therapy. It is important to note that 25% of patients with a partial colectomy may have unsatisfactory results leading to other complications such as adhesions, bleeding, thrombosis, and adjacent organ damage (Christiansen and Rasmussen, 1996). Chronic constipation has an impact on a patient's HRQoL, and has economic consequences for both the affected individual and for society (Dennison et al., 2005; Wald et al., 2007).

A review by Dennison et al. found that individuals with constipation had lower HRQoL than those without the condition (Dennison et al., 2005). Constipation symptom measures include the Gastrointestinal Symptom Rating Scale, the Elderly Bowel Symptom Questionnaire, and the Patient Assessment of Constipation Symptoms (PAC-SYM) questionnaire (Dennison et al., 2005). The PAC-SYM questionnaire is one component of the Patient Assessment of Constipation (PAC) tool; the other component being the PAC-Quality of Life (PAC-QOL) questionnaire, which is a constipation-specific measure of HRQoL. The two components are complementary and may be used separately or together (Frank et al., 1999; Marquis et al., 2005).

Prucalopride belongs to the chemical class of dihydrobenzofuran-carboxamide derivatives with potent enterokinetic activity (SmPC Prucalopride^1^). Serotonin (5-HT) signaling in the gastrointestinal tract is known to regulate a range of functions including motility and, consequently, efforts have been made to develop selective 5-HT_4_ agonists for treating disorders in which gastrointestinal motility is impaired, such as constipation (Schiller, 2004; Galligan and Vanner, 2005). The efficacy and safety of prucalopride has been investigated in three pivotal, identically designed, double-blind, placebo-controlled, phase 3, randomized clinical trials (NCT00483886, NCT00485940, and NCT00488137) (Camilleri et al., 2008; Quigley et al., 2009; Tack et al., 2009). European Medicines Agency regulatory approval was based on the results of these trials. In Europe, the Summary of Product Characteristics (SmPC) for prucalopride states: “prucalopride (a selective, high affinity 5-hydroxytryptamine receptor 4 [5-HT_4_] agonist) is indicated for symptomatic treatment of chronic constipation in women in whom laxatives fail to provide adequate relief” (SmPC Prucalopride). The licensed prucalopride dose is 2 mg once daily; in women aged over 65 years, the recommended starting dose is 1 mg once daily, increasing to 2 mg once daily, if required. According to the SmPC, if once-daily prucalopride is not effective after 4 weeks of treatment, the patient should be re-examined and the benefit of continuing treatment reconsidered.

The constipation-specific PAC-SYM and PAC-QOL measures were used in clinical trials of prucalopride. Parker et al. examined the extent to which these measures can be mapped to two preference-based generic instruments, the 5-Dimension EuroQol questionnaire (EQ-5D) and the Short Form-6 Dimension questionnaire (SF-6D), to generate robust and reliable utility estimates (Parker et al., 2011). PAC-QOL scores generated in the pivotal clinical trials of prucalopride were converted into utility values estimated using the EQ-5D. The 36-Item Short Form Survey (SF-36) was also collected in the clinical trials and these data were used to generate SF-6D estimates for comparative purposes. The mean utility estimate at baseline for chronic constipation, based on the EQ-5D data was 0.813 [standard deviation (SD): 0.175], and 0.723 (SD: 0.126) when based on the SF-6D data.

An analysis by Tack et al. showed that prucalopride 2 mg once daily for 12 weeks alleviates common constipation symptoms in women in whom laxatives had failed to provide adequate relief (Tack et al., 2014). Given its proven effectiveness when laxatives have failed to provide adequate relief (Camilleri et al., 2008; Quigley et al., 2009; Tack et al., 2009) the cost of prucalopride may be justified by a favorable incremental cost-effectiveness ratio (ICER). An economic analysis reviewed by the National Institute for Health and Care Excellence (NICE) showed that prucalopride was cost-effective in the UK, with the average cost per quality-adjusted life-year (QALY) gained being € 19,728 (~£ 15,700) for prucalopride relative to continued laxative treatment (NICE technology appraisals TA211, 2010).

The objective of the present study was to assess the cost-effectiveness of prucalopride vs. continued laxative treatment for chronic constipation in patients in the Netherlands in whom laxatives, over an extended period of time, had failed to provide adequate relief.

## Methods

In this study, a Markov model was used to estimate the cost-effectiveness of prucalopride in a cohort of patients with chronic constipation in the Dutch healthcare setting in 2011, when clinical data were generated in the pivotal trials of prucalopride. Data sources included published clinical trials, published Dutch price/tariff lists, and national population statistics. The inclusion criteria were the same as for the clinical trials of prucalopride, and effectiveness was expressed as QALYs. The model included relevant economic measures such as resource utilization patterns associated with outpatient and inpatient care for patients with chronic constipation. The primary perspective of the study was that of the Dutch payer rather than a broader societal perspective (Richtlijnen voor farmaco-economisch onderzoek, 2006). Costs in the model were expressed in 2011 euros.

The model structure and the associated treatment patterns, data sources, and assumptions were validated at a Delphi panel meeting in Utrecht, Netherlands on 25 March 2010. The panel consisted of seven gastrointestinal specialists and four primary care practitioners (PCPs) working in the Netherlands who had experience of treating chronic constipation. Sensitivity analyses and scenario analyses explored uncertainty in data sources, structure, and assumptions.

### Model design

**Figure 1:**
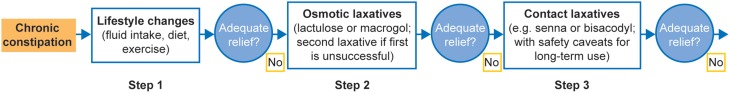
**Chronic constipation treatment pathway in the Netherlands**.

Dutch treatment guidelines take a step-by-step approach to the management of patients with chronic constipation (Figure 1) (Nederlands Huisartsen Genootschap^2^). Initially, diet and exercise are recommended, followed by use of laxatives. However, individuals who have had chronic constipation for many years and who fail to achieve symptomatic relief with laxatives have limited treatment options and therefore are often switched from one laxative to another many times, as established at the 2010 Delphi panel meeting (data on file). Dutch guidelines do not provide recommendations for switching treatments in primary care after step 3 (Figure 1) so patients will continue to be switched to another laxative according to the standard of care defined by the Nederlands Huisartsen Genootschap—Dutch Society of General Practitioners (Diemel et al., 2010).

In the UK, NICE recommendations essentially follow the same treatment pathway, advocating a switch or the addition of a second laxative from a different class if the first laxative does not provide adequate relief from constipation at the maximum recommended and tolerated dose (NICE technology appraisals TA211, 2010). NICE considered both the drug and other medical costs of treating patients with chronic constipation when laxatives fail to provide adequate relief, and agreed that these costs could be reduced by using prucalopride. Because of similarities in the treatment pathways between the Netherlands and the UK, the Dutch model could have been based on a similar design, population, comparator, and assumptions to the UK model. However, this UK model included only drug costs, whereas Dutch health economic guidelines (Richtlijnen voor farmaco-economisch onderzoek, 2006) for the analysis of treatment for a chronic disease require the inclusion of not only drug costs but all direct medical costs, as well as assessment over a long time horizon. Therefore, in the present study, a Markov model was developed for the Netherlands that included these additional costs and also an extended time horizon (1 year in the base case analysis and up to 3 years in the scenario analysis). A Markov model was chosen because this type of model is considered more transparent than the stochastic model used in the UK, and is widely accepted by the Dutch health authorities.

**Figure 2:**
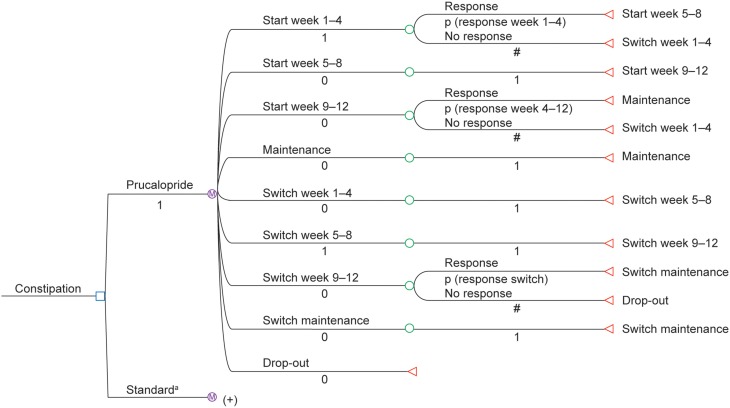
**Structure of the Markov model (p, probability; #, 1—probability)**. ^a^ (+) The structure for continued laxative treatment is the same as the structure for prucalopride.
Start week 1–4: prucalopride (or continued laxative) treatment was started.Start week 5–8: prucalopride (or continued laxative) treatment was continued after 4 weeks.Start week 9–12: prucalopride (or continued laxative) treatment was continued after 8 weeks.Maintenance: patient was responsive to prucalopride (or continued laxative) treatment after 12 weeks.Switch week 1–4: a new medication was started after failure of the initial treatment; the initial medication was stopped. The new treatment consisted of laxatives, in line with the 2010 Delphi panel recommendations (described in detail in the Data Sources Section).Switch week 5–8: the new treatment was continued after 4 weeks.Switch week 9–12: the new treatment was continued after 8 weeks.Switch maintenance: the patient was responsive to the new treatment.Drop-out: patients dropped out of the model if the new (post-switch) treatment failed, and remained drop-outs for the rest of the time horizon of the model. The treatment for these patients also consisted of laxatives, in line with the 2010 Delphi panel recommendations.

Markov techniques were used to model the clinical and economic outcomes in patients receiving prucalopride or continued laxative treatment, allowing long-term analysis of chronic constipation beyond the follow-up period in the pivotal clinical trials. Figure 2 shows the structure of the model. The first branch point was a decision node: prucalopride vs. continued laxative treatment (standard care), and the structure of both these arms was identical. The number of transitions allowed per cycle is shown in Figure 2. The cycle length chosen for the model was 1 month, which closely approximates the intervals of measurement in the three pivotal phase 3 prucalopride randomized clinical trials (Camilleri et al., 2008; Quigley et al., 2009; Tack et al., 2009). The follow-up time (analytical horizon) used in the model was 1 year (base-case analysis).

The sequence of the initial states (weeks 1–4, 5–8, and 9–12) was based on the design of the pivotal phase 3 trials (Camilleri et al., 2008; Quigley et al., 2009; Tack et al., 2009), in which response to prucalopride and its comparator was reported after week 4 and week 12. The model included a stopping rule that was not applied in the clinical trials: if patients did not respond within 4 weeks, the initial prucalopride (or continued laxative) treatment was discontinued and patients were switched to another laxative. This was consistent with the cost-effectiveness model for the UK and the NICE recommendation.

### Model assumptions

- The model did not include mortality: the time horizon of the model in the base-case analysis was limited to 1 year and no incremental difference in mortality was expected between treatment groups.
- The probability of a response to a new treatment following failure of the previous one was assumed to be similar to that for the previous treatment.
- The treatment patterns following failure of an initial treatment were assumed to be similar to the initial pattern.
- Discontinuation during maintenance with prucalopride or continued laxative treatment was not included.
- Modeling was performed using responses across both sexes.
- Side effects of treatments were not included in the model because they were minor, transient, and assumed not to impact on resource utilization from a payer's perspective.
- “Normal bowel function” was used to measure the effectiveness of treatment.

### Population and comparison

The population comprised patients with long-term functional chronic constipation inadequately relieved by laxatives, and the model used data from the populations included in the pivotal prucalopride studies (Camilleri et al., 2008; Quigley et al., 2009; Tack et al., 2009). Altogether, the three studies included 1977 adults with similar baseline characteristics: 90% were women and 69% had had constipation for 20 years or longer. Although the trial populations included men, the Committee for Medicinal Products for Human Use review concluded that, based on its risk–benefit balance, prucalopride should be licensed for symptomatic treatment of chronic constipation only in women (European Medicines Agency^3^). NICE also concluded that prucalopride was clinically effective in providing relief to women with laxative-refractory chronic constipation (NICE technology appraisals TA211, 2010). Therefore, this cost-effectiveness study included a scenario analysis for female patients older than 18 years.

### Time horizon

Dutch guidelines recommend analysis of long-term clinical effectiveness in the evaluation of healthcare technologies (Richtlijnen voor farmaco-economisch onderzoek, 2006); the base-case analysis therefore assumed a 1-year follow-up period. The pivotal clinical trials for prucalopride covered a period of 12 weeks; however, long-term observational data (Camilleri et al., 2010) concerning patient satisfaction were collected, which showed that, between week 12 and week 52, patients experienced no significant changes in the efficacy of prucalopride. These data were used to extend the time frame in the model to 12 months.

### Effectiveness and economic outcomes

The primary analysis was a cost–utility analysis in line with Dutch health economic guidelines (Richtlijnen voor farmaco-economisch onderzoek, 2006), and the primary outcome measure was incremental cost per QALY gained.

Medical costs reflected the resource utilization associated with treatment of chronic constipation and its complications. The costs of over-the-counter laxatives were not included, because individuals with chronic constipation are treated within the medical setting (e.g., by their primary care physician [PCP] or a specialist), and therefore reimbursed laxatives are prescribed. A scenario analysis was performed including indirect costs due to productivity loss derived from international studies, as no Dutch data on these costs were available.

Because the time horizon of the base-case analysis was 1 year, discounting was not applied. A 3-year scenario analysis was conducted, however, using discount rates of 4% for economic outcomes and 1.5% for clinical outcomes, in line with Dutch guidelines (Richtlijnen voor farmaco-economisch onderzoek, 2006).

### Sensitivity and scenario analysis

Sensitivity analyses were performed on:

- Response to prucalopride at week 4
- Response to prucalopride at week 12
- Diagnose Behandel Combinatie (DBC) hospitalization
- DBC specialist consultation

Scenario analyses were performed on:

- Response for women only
- Response based on the PAC-QOL patient satisfaction subscale
- Excluding the stopping rule at 4 weeks
- Excluding cost of complications
- Using an average daily dose of 1.5 mg for prucalopride rather than the 2 mg daily dose used in the base-case analysis
- Including indirect costs due to lost productivity
- Excluding transportation costs
- Using hospital costs based on daily costs and length of stay rather than the Dutch diagnosis treatment code list fixed price
- A time horizon of 3 years instead of 1 year
- Including the effect of mortality
- Inflating costs to 2014 values

## Data sources

### Clinical data

Tables 1–**4** provide an overview of the clinical data for the Markov model, based on pooled information from the pivotal phase 3 trials; these data were also used in the UK cost-effectiveness model (Camilleri et al., 2008; Quigley et al., 2009; Tack et al., 2009). The primary cost-effectiveness analysis was based on the intent-to-treat population. Placebo response was used as a proxy for response to laxatives in patients with chronic constipation; this was supported by a meta-analysis that showed poor evidence for sustained benefit with laxatives (Jones et al., 2002).

**Table 1:** **Number (%) of patients with three or more spontaneous complete bowel movements per week**.

**Time point**	**Placebo (*N* = 645; *F* = 580) *n* (%)**	**Prucalopride 2 mg (*N* = 640; *F* = 566) *n* (%)**	**Prucalopride 4 mg (*N* = 639, *F* = 558) *n* (%)**
**WEEKS 1–12**			
All	73 (11.3)	151 (23.6)^*^	158 (24.7)^*^
Female	62 (10.7)	138 (24.4)^*^	136 (24.4)^*^
**WEEKS 1–4**			
All	68 (10.5)	178 (27.8)^*^	192 (30.0)^*^
Female	58 (10.0)	166 (29.3)^*^	169 (30.3)^*^

*Pooled data from the intent-to-treat population in the pivotal prucalopride clinical trials (Camilleri et al., 2008; Quigley et al., 2009; Tack et al., 2009) (F, female)*.

* *P < 0.001 vs. placebo (pairwise comparison)*.

The pivotal trials demonstrated that men respond to prucalopride in a similar way to women; however, given the small number of men in the trials, no significant difference in efficacy was demonstrated between prucalopride and placebo. Modeling was performed using responses across both sexes, because the utility mapping applied to the entire trial population. Because the utilities in this model are based on empirical mapping between PAC-QOL and EQ-5D for the entire trial population (see the Utilities Section), we decided to use the efficacy data from the entire trial population in the base-case analysis. A separate scenario analysis was performed to consider women only (the target population) in order to ascertain the uncertainty associated with the assumption that men would respond similarly to women. One recent publication has shown that the efficacy of prucalopride is similar in the intent-to-treat population and the target population (Tack et al., 2013), while the results of another clinical trial have shown that efficacy is similar in men and women (Ke et al., 2012).

The base-case analysis was based on the primary clinical outcome from the pivotal trial. Response was defined as the proportion of patients who achieved “normal bowel function” within the 4-week and 12-week periods; normal bowel function was defined as a mean of three or more spontaneous and complete bowel movements per week. The response rate after 4 weeks was 10.5% for placebo, 27.8% for prucalopride 2 mg once daily, and 30.0% for prucalopride 4 mg once daily, and after 12 weeks was 11.3% for placebo, 23.6% for prucalopride 2 mg once daily, and 24.7% for prucalopride 4 mg once daily (Table 1). The base-case analysis was based on prucalopride 2 mg once daily and so data for this dose from the clinical trials were used.

A scenario analysis used the PAC-QOL patient satisfaction subscale (Table 2) as the primary quality of life parameter. One point on this subscale was the predefined threshold for a clinically meaningful response; this is twice the minimal important difference (0.5) (Dubois et al., 2010).

**Table 2:** **Number (%) of patients with at least 1 point improvement on the satisfaction subscale of the Patient Assessment of Constipation Quality of Life instrument**.

**Time point**	**Placebo (*N* = 645)**		**Prucalopride 2 mg (*N* = 640)**		**Prucalopride 4 mg (*N* = 639)**	
	***N***	***n* (%)**	***N***	***n* (%)**	***N***	***n* (%)**
Week 12^a^	618	137 (22.2)	621	273 (44.0)^*^	603	261 (43.3)^*^
Week 4^a^	605	129 (21.3)	598	271 (45.3)^*^	588	270 (45.9)^*^

*Pooled data from the intent-to-treat population in the three pivotal prucalopride clinical trials (Camilleri et al., 2008; Quigley et al., 2009; Tack et al., 2009)*.

a *Last observation carried forward*.

* *P < 0.001 vs. placebo (pairwise comparison)*.

Probabilities of response after switching to another treatment were also included in this study. These data were not available in the published literature; therefore, probabilities estimated by the 2010 Dutch Delphi panel were used (data on file) (Table 3).

**Table 3:** **Delphi panel estimates of the probabilities of a response after switching treatment to prucalopride, treatment practices after treatment failure, and complications in patients after treatment for chronic constipation or a switch in treatment to prucalopride (PCP, primary care physician)**.

	**Probability (%)**		
**TREATMENT SWITCH**			
Response after switching treatment	65.4		
**TREATMENT PRACTICE AFTER FAILURE**			
Referral to specialist by PCP	28.2		
Hospitalization	4.9		
No referral or hospitalization	66.9		
**Probability of complications**	**Weeks 1–4**	**Weeks 5–12**	**Switch**
Hemorrhoids	19.0	22.0	12.0
Anal fissures	7.0	11.0	6.0
Fecal incontinence	8.0	7.0	6.0
External peri-anal thrombosis	7.0	9.0	2.0
Rectal prolapse	3.4	5.0	0.8
Fecal impaction	14.1	15.4	3.8

Table 3 also provides the 2010 Dutch Delphi panel's estimates for probabilities of complications of constipation. Singh et al. showed the probability of such complications using a California Medicaid database (Singh et al., 2007), reporting a 2.05% probability of fecal impaction; the probability reported by the 2010 Dutch Delphi panel was 14.1% at the start of treatment. However, the Singh et al. study related to patients newly diagnosed with constipation in a single state in the USA; therefore the Dutch Delphi estimates were considered more appropriate than the Singh data for a population in the Netherlands with chronic constipation. The estimates made by the Delphi panel were adjusted to monthly probabilities in order to correspond with the cycle time of the model.

### Utilities

Empirical mapping of PAC-QOL scores to EQ-5D scores provided data on utilities for patients using prucalopride or continued laxative treatment. Pooled data from the pivotal phase 3 studies provided response and overall PAC-QOL outcomes (Camilleri et al., 2008; Quigley et al., 2009; Tack et al., 2009). Parker et al. used statistical methods to identify a mapping function to assess the extent to which changes in generic HRQoL can be “explained” by more condition-specific measures, these were used in the UK cost-effectiveness model (Parker et al., 2011; Tack et al., 2014). A relationship was determined between PAC-QOL and EQ-5D.

The model developed by Parker et al. stratifies patients as responders and non-responders to prucalopride (or continued laxative) treatment. Therefore, PAC-QOL scores were determined separately for responders and non-responders. Table 4 provides a list of utilities for the Markov model based on this mapping study. These are in line with the utilities used in a previous cost-effectiveness model comparing macrogol and lactulose in the treatment of patients with chronic constipation: 0.06 monthly utility for symptomatic states and 0.07 monthly utility for well-controlled states, corresponding to annual utilities of 0.72 and 0.84, respectively (Taylor and Guest, 2010).

**Table 4:** **Utilities for the different health states in the model**.

	**Utility**
**PRUCALOPRIDE**	
Starting treatment (weeks 1–4)	0.786
Continuing treatment after 4 weeks (weeks 5–8)	0.813
Continuing treatment after 8 weeks (weeks 9–12)	0.813
Responders to prucalopride (> 12 weeks)	0.890
**CONTINUED LAXATIVE TREATMENT**	
Starting treatment (weeks 1–4)	0.781
Continuing treatment after 4 weeks (weeks 5–8)	0.805
Continuing treatment after 8 weeks (weeks 9–12)	0.805
Responders to continued laxative treatment (> 12 weeks)	0.879
**SWITCH**	
Starting treatment with a new treatment (weeks 1–12)	0.784
Responders to switch treatment (> 12 weeks)	0.879
Drop-out	0.784

For patients in whom initial treatment failed, or those who were switched to another medication, the same average baseline utility based on the placebo and prucalopride arms was used. This is a conservative assumption since, if a patient is not improving, their utility could decline to the placebo level.

### Resource utilization

The medication costs for continued laxative use and for other treatments were derived from the distribution of the various types of medication and their dosages, as reported by the 2010 Delphi panel, and published Dutch list prices (Farmacotherapeutisch Kompas).

The non-drug resource utilization for each health state was programmed as a monthly cost. The following were included: the management of chronic constipation; the additional resource utilization associated with a change of therapy after a failure; and the additional resource utilization resulting from complications.

Because published data on such resource use were limited, the 2010 Delphi panel's estimates were used (Tables 5, 6).

**Table 5:** **Resource utilization of patients with chronic constipation who did not obtain adequate relief from laxatives, determined by the Delphi process^a^ (GI, gastrointestinal; PCP, general practitioner)**.

	**Number per month**				
	**Weeks 1–4**	**Weeks 5–12**	**Maintenance**	**Switch weeks 1–4**	**Switch maintenance**
**CONSULTATIONS**					
Specialist consultation	0.6	0.6	0.4	0.8	0.3
Specialist telephone call	0.3	0.6	0.4	0.4	0.1
PCP consultation	0.9	1.0	0.7	0.7	0.6
PCP telephone call	0.6	1.1	0.5	0.3	0.3
Nurse consultation	0.2	0.2	0.2	0.2	0.1
Dietician consultation	0.1	0.2	0.3	0.2	0.1
**PROCEDURES**					
Colonoscopy/gastroscopy	0.6	0.5	0.4	0.4	0.2
Digital rectal examination	0.8	0.6	0.4	0.6	0.3
Anoscopy/proctoscopy	0.2	0.5	0.1	0.2	0.2
Blood tests	0.8	0.9	0.5	0.0	0.0
Radiograph	0.2	0.7	0.5	0.3	0.1
GI transit	0.2	0.0	0.0	0.2	0.1
Evacuation/Fleet® enema	0.3	0.6	0.5	0.2	0.0

a *As per the Delphi survey, resource use applies to a patient population with chronic constipation not adequately relieved by laxatives and seeking medical care for their continuing condition and likely complications*.

**Table 6:** **Resource use related to complications per patient with chronic constipation who did not obtain adequate relief from laxatives, determined by the Delphi process^a^ (PCP, primary care physician)**.

	**Number of consultations per month**		**Proportion of cases with hospitalization**	
	**PCP**	**Specialist**	**%**	**Duration of stay (days)**
Hemorrhoids	2.2	1.5	1.7	1.0
Anal fissures	2.3	1.0	1.7	3.0
Fecal incontinence	1.8	1.6	8.9	4.5
External peri-anal thrombosis	2.0	1.1	5.6	1.7
Rectal prolapse	1.2	1.7	36.7	5.2
Fecal impaction	1.8	1.2	16.2	3.0

a *As per the Delphi survey, resource use applies to a patient population with chronic constipation not adequately relieved by laxatives and seeking medical care for their continuing condition and likely complications*.

### Costing

The following costs were included in the model (Table 7).

- Annual drug costs were derived from published Dutch list prices (Farmacotherapeutisch Kompas^4^) based on recommendations in the Dutch Costing Manual (Handleiding voor kostenonderzoek, 2010). The cost of continued laxative treatment was derived from a weighted average based on distribution recommended by the 2010 Delphi panel. For prucalopride, the base-case analysis was based on 2 mg daily for a maximum period of 220 days per year according to clinical data. A scenario analysis was performed on 1.5 mg prucalopride daily, assuming a 50% divide between 1 and 2 mg daily dose and similar effectiveness in a daily practice setting.
- A stopping rule was applied as follows: if once-daily prucalopride was not effective after 4 weeks of treatment according to the SmPC, prucalopride was discontinued. The drug costs and Dutch prescription rule (“receptregel”) included 6% value added tax; the prescription rule means that there is a fixed fee for each prescription dispensed by the pharmacist.
- Based on long-term trial data, the average annual cost assumed that, in clinical practice, the average duration of use of prucalopride in the maintenance phase was 130 days per year in the first year and 220 days in the second and third years (NICE technology appraisals TA211, 2010). The maximum annual period of treatment was 220 days and therefore, after the initial period of 3 months (90 days), 130 treatment days were left in the first year. This was consistent with the UK cost-effectiveness model.
- PCP consultation tariffs were derived from the Dutch Costing Manual.
- Costs of outpatient visits to specialists were based on the Dutch Diagnosis Behandel Combinatie (diagnosis treatment code) list (which assumes a fixed overall cost for a specialist consultation). The analysis was based on a minimum value of € 195.89.
- Hospitalization costs were based on the Dutch diagnosis treatment code list (which assumes a fixed overall cost for a hospitalization). The analysis was based on a minimum value of € 3102 (in a scenario analysis, costs were calculated by the product of length of stay and daily cost).
- Non-medical direct costs were calculated based on the following average distances: 1.1 km to the PCP's surgery, 7.0 km to the specialist and to the hospital, and 1.7 km to the dietitian. These distances were multiplied by cost per kilometer of € 0.20, which was similar whether driving or taking public transport (year of costing 2009, inflated to 2011). The base-case analysis was based on transportation costs only, excluding parking costs. A scenario analysis also included additional costs of € 3.00 for parking, if driving.

**Table 7:** **Costing information included in the Markov model (CBS, Centraal Buro Satistiek; DBC, Diagnose Behandel Combinatie [Dutch diagnosis treatment code]; PCP, primary care physician; NZa, Nederlandse Zorgautoriteit [Dutch health authority]**.

	**Dose**	**First pack (28 days)**	**After 28 days**	**Data source**		**Costing year**
Prucalopride	1 mg	€ 1.95 per day	€ 1.62 per day	Shire-Movetis		2010
	2 mg	€ 2.77 per day	€ 2.49 per day	Shire-Movetis		2010
	**Pack size**	**Cost per pack**	**Distribution start (switch)^a^**	**Daily dose**	**Daily cost**	**Costing year**
**SOURCE: FARMACOTHERAPEUTISCH KOMPAS**						
Movicolon® sachets	1 sachet	€ 0.32	42% (27%)	1.5 sachet	€ 0.48	2010
Bisacodyl	5 mg	€ 0.11	8% (17%)	7.5 mg	€ 0.17	2010
Forlax®	10 g	€ 0.61	15% (7%)	10 g	€ 0.61	2010
Metamucil®	1 sachet	€ 0.30	18% (23%)	2 sachets	€ 0.60	2010
Lactulose	10 g	€ 0.23	17% (0%)	10 mg	€ 0.23	2010
Magnesium oxide	500 mg	€ 0.03	0% (26%)	3.5 g	€ 0.21	2010
Prescription rule		€ 6.35				
	**Cost/unit**			**Data source**		**Costing year**
PCP	€ 28.00			Costing manual		2009
Specialist	€ 72.00			Costing manual		2009
PCP telephone call	€ 14.00			Costing manual		2009
Nurse	€ 10.00			Costing manual		2009
Nurse telephone call	€ 0.00			Costing manual		2009
Dietician	€ 27.00			Costing manual		2009
DBC: polyclinic for constipation	€ 195.89			DBC 1337101^b^ (NZa tarieven)		2010
DBC: hospitalization for constipation	€ 3102.00			DBC 141508 (NZa tarieven)		2010
	**Inflation rate**			**Data source**		**Costing year**
2009	1.2%			CBS		2009
2010	1.3%			CBS		2010

a *Distribution of laxatives at start (and after switching) of therapy*.

b *See Supplementary Material*.

### Indirect costs

Indirect costs were calculated using the methodology described in the Dutch Manual for Costing and recent economic data from the main Dutch national statistical data source to reflect the patient population treated (Handleiding voor kostenonderzoek, 2010). As mentioned earlier, indirect costs were not included in the base-case analysis owing to lack of Dutch data on lost productivity. A scenario analysis was therefore performed using international data. A survey in 2007 showed that 12% of respondents who worked or attended school reported missing a mean of 2.4 days per month from work or class because of chronic constipation symptoms (Johanson and Kralstein, 2007). The mean cost per hour lost was € 26.99 and the mean annual number of working hours lost was 1540 (Handleiding voor kostenonderzoek, 2010). The overall pooled percentage of patients aged between 18 and 65 years was 87.0% (European Medicines Agency).

## Results

### Base-case analysis

The base-case analysis indicated that the total cost of treatment over a period of 1 year with prucalopride was € 2511 per patient, compared with € 2446 per patient for continued laxative treatment (Table 8). The average patient treated with prucalopride gained 0.833 QALYs, compared with 0.826 for those on continued laxative treatment. The ICER for prucalopride treatment relative to continued laxative treatment was € 9015 per QALY gained.

**Table 8:** **Results of the base-case analysis indicating the costs, QALYs, and ICER associated with prucalopride vs. continued laxative treatment for chronic constipation (ICER, incremental cost-effectiveness ratio; QALY, quality-adjusted life-year)**.

	**Costs**	**QALYs**	**ICER**
Prucalopride	€ 2511	0.833	
Continued laxative treatment	€ 2446	0.826	
Difference	€ 65	0.007	€ 9015

### Scenario analyses

The results of the scenario analyses are presented in the Supplementary Material.

### Sensitivity analyses

In addition to these scenario analyses, the sensitivity of the base-case results to a range of key parameters was assessed.

#### One-way sensitivity analyses

**Figure 3:**
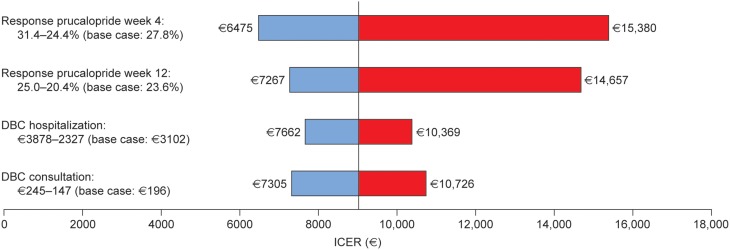
**Tornado diagram of incremental cost-effectiveness ratios of prucalopride relative to continued laxative use determined by one-way sensitivity analyses (DBC, Diagnose Behandel Combinatie [Dutch diagnosis treatment code]; ICER, incremental cost-effectiveness ratio)**.

Results of one-way sensitivity analyses (shown in Figure 3, with detailed results in Table 9) showed that the model is sensitive to the response to prucalopride. The ICER varied from € 6475 to 15,380 per QALY gained for response at week 4, and from € 7267 to 14,657 per QALY gained for response at week 12. The analyses showed that the model was moderately sensitive to Dutch diagnosis treatment code values. The ICER varied from € 7662 to 10,369 per QALY gained for hospitalization, and from € 7305 to 10,726 per QALY gained for consultation.

**Table 9:** **Costs and QALYs associated with a response to prucalopride determined by sensitivity analyses (CI, confidence interval; DBC, Diagnose Behandel Combinatie [Dutch diagnosis treatment code]; ICER, incremental cost-effectiveness ratio; QALY, quality-adjusted life-year)**.

	**Costs (€)**	**QALYs**	**ICER**
**RESPONSE TO PRUCALOPRIDE (4 WEEKS) (BASE CASE: 27.8%)**			
Minimum: 24.4% (2.5% CI)			
Prucalopride	2525	0.831	
Continued laxative treatment	2446	0.826	
Difference	79	0.005	15,380
Maximum: 31.4% (97.5% CI)			
Prucalopride	2501	0.835	
Continued laxative treatment	2446	0.826	
Difference	56	0.009	6475
**RESPONSE TO PRUCALOPRIDE (12 WEEKS) (BASE CASE: 23.6%)**			
Minimum: 20.4% (2.5% CI)			
Prucalopride	2528	0.832	
Continued laxative treatment	2446	0.826	
Difference	82	0.006	14,657
Maximum: 25.0% (97.5% CI)			
Prucalopride	2503	0.834	
Continued laxative treatment	2446	0.826	
Difference	57	0.008	7267
**DBC: HOSPITALIZATION**			
Minimum: 25% lower			
Prucalopride	2452	0.833	
Continued laxative treatment	2377	0.826	
Difference	75	0.007	10,369
Maximum: 25% higher			
Prucalopride	2570	0.833	
Continued laxative treatment	2514	0.826	
Difference	55	0.007	7662
**DBC: CONSULTATION SPECIALIST**			
Minimum: 25% lower			
Prucalopride	2146	0.833	
Standard care	2068	0.826	
Difference	77	0.007	10,726
Maximum: 25% higher			
Prucalopride	2876	0.833	
Standard care	2823	0.826	
Difference	53	0.007	7305

#### Probabilistic sensitivity analysis

A probabilistic sensitivity analysis was performed to explore the uncertainty in the estimates of the input parameters. Resource use quantities were assumed to follow gamma distributions, while utilities and probabilities were assumed to follow beta distributions (Briggs, 2000; Briggs et al., 2002). The analysis was based on 5000 patients. Empirically, a plateau to 1 was reached from 4000 onward. The standard deviation did not change from 4000 cycles onward. The ICER was € 8892 per QALY gained compared with € 9015 in the base-case analysis.

**Figure 4:**
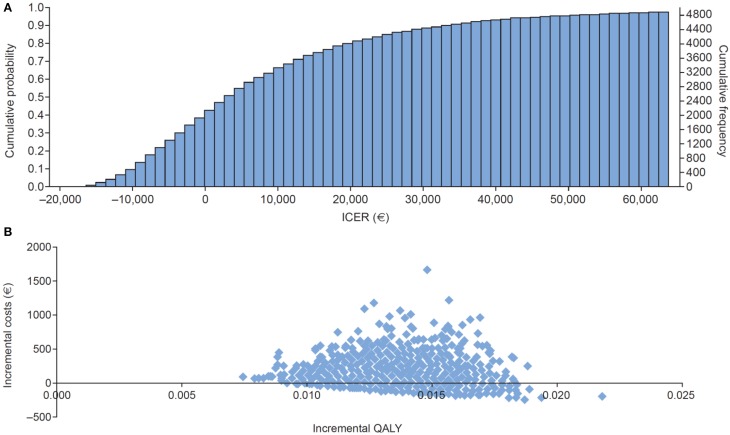
**(A)** Cost-effectiveness acceptability plot for the base-case analysis. **(B)** Scatter plot showing the cost-effectiveness estimates for the base-case analysis (ICER, incremental cost-effectiveness ratio; QALY, quality adjusted life-year).

The cost-effectiveness acceptability plot shown in Figure 4A reflects the probability that each treatment is the most cost-effective treatment considered in the analysis at a range of different threshold ICER values. The results indicate there is a greater than 80% probability that prucalopride is more cost-effective than continued laxative treatment, if Dutch society is willing to pay at least € 20,000 per QALY gained. Figure 4B shows the incremental outcomes for costs and QALYs resulting from the probabilistic sensitivity analysis.

## Discussion and conclusion

The base-case model revealed that prucalopride 2 mg once daily is a cost-effective option for patients with chronic constipation in the Netherlands, costing € 9015 per QALY gained relative to continued laxative treatment. Extensive scenario analyses and sensitivity analyses underlined the robustness of the base-case result. In these analyses, key model parameters were varied substantially. In the scenario analysis examining the response in women only, the label target population, the ICER decreased from € 9015 to 7773 per QALY gained.

In this context, it is noteworthy that this cost-effectiveness model included information from three large international pivotal trials (Camilleri et al., 2008; Quigley et al., 2009; Tack et al., 2009), showed a consistent response for multiple endpoints, and involved a trial population similar to the patient population in clinical practice.

Defining the response in terms of frequency of bowel movements does not encompass all aspects of efficacy. For this reason, patient satisfaction reflects the efficacy of prucalopride in clinical practice better than the primary outcome measure in the pivotal clinical trials, which was used in the base-case analysis (i.e., three or more spontaneous complete bowel movements per week). The predefined primary HRQoL endpoint dimension of the PAC-QOL was therefore used in a scenario analysis, resulting in a decrease in ICER from € 9015 to 5197 per QALY gained. The clinical relevance of satisfaction in chronic constipation has been shown in a study by Müller-Lissner et al. (2010).

Owing to a lack of solid Dutch data, indirect costs were excluded from the base-case model. A scenario analysis included lost productivity based on international data on working days lost, resulting in a decrease in ICER from € 9015 to 5228 per QALY gained. Hence, the base-case analysis was conservative.

The use of a Delphi panel to establish resource utilization data when no such data exist is accepted methodology according to Dutch health economic guidelines (Richtlijnen voor farmaco-economisch onderzoek, 2006). The proportion of complications in patients with chronic constipation was also estimated by the Delphi panel owing to a lack of data. Exclusion of such complications from the analysis resulted in an increase in ICER from € 9015 to 12,216 per QALY gained; therefore, prucalopride remained cost effective when complications were not considered.

The time horizon of the model was only 1 year because of a lack of long-term data. However, a scenario analysis over a 3-year period confirmed the robustness of the outcome: the ICER decreased from € 9015 to 4436 per QALY gained, illustrating that the 1-year results probably underestimate the cost-effectiveness of prucalopride.

In the Netherlands, the threshold for a drug to be considered cost-effective varies from € 10,000 to 80,000 per QALY gained, depending on the severity of the disease. Extensive scenario and sensitivity analyses underlined the robustness of the base-case cost-effectiveness of prucalopride.

In Europe, registration of prucalopride is for women for whom laxative treatment has failed, whereas the pivotal trials included both men and women, and the mapping of utilities was performed using the clinical outcomes for this total population. A scenario analysis considering only women confirmed the robustness of this approach, because the ICER decreased only from € 9015 to 7773 per QALY gained. The base-case analysis was therefore conservative.

In the UK cost-effectiveness model, the patient population was created by bootstrapping (re-sampling) a repeated random draw from the 1462 patients in the pivotal trials by age category, using the age and corresponding baseline severity (EQ-5D utility score) distributions defined by these 1462 patients. A cohort of 38,650 patients was found to be sufficient to reduce the variability (sampling error) and provide a stable outcome in the model, and therefore this formed the basis for the cost–utility analysis of the use of prucalopride in the Netherlands. It is important to note that the bootstrap was performed only to create the model's population, and was not used in the regression analyses or in the mapping process, for which actual observations were used. This population allows the analysis of all women (18 years or older) as well as of other subgroups.

There were a number of reasons to develop a Markov model for the Netherlands. Dutch health economic guidelines require the inclusion of all medical costs, whereas the UK model considered only drug costs. Another reason for choosing a Markov model was to enable long-term analysis beyond the clinical trial period, in line with Dutch guidelines for pharmacoeconomic research (Richtlijnen voor farmaco-economisch onderzoek, 2006). Increasing the time horizon to 3 years instead of 1 year resulted in a decrease in the ICER; consequently the 1-year time horizon may underestimate the true cost-effectiveness of prucalopride.

The current study used placebo response as a proxy for the response to laxatives in patients with chronic constipation. An alternative option would have been to use another laxative as a comparator. In this case it was decided not to use that approach, as there is a lack of clinical evidence for sustained benefit with laxatives (Jones et al., 2002). Nevertheless, future studies may wish to consider this.

The published pharmacotherapeutic report on prucalopride by the Medicinal Products Reimbursement Committee (CFH) indicated a negative opinion in their evaluation (approved 26 November 2012). The main criticism of the CFH was the association between the defined patient population and the clinical evaluation. The report explained that it was unclear whether the patients included in the studies were refractory to optimum doses of standard laxatives. Nevertheless, in clinical practice, physicians will generally take into account the balance between efficacy and tolerance in each patient that they treat. Therefore, healthcare practitioners will not always prescribe the maximum recommended dose, and the positive cost-effectiveness benefits of prucalopride in the Netherlands should still be valid.

Besides the positive cost-effectiveness benefits of prucalopride for the Dutch payer and society, prucalopride also has a positive impact on patients with chronic constipation. Prucalopride treatment increases not only the number of spontaneous complete bowel movements but also patient satisfaction and HRQoL.

Finally, the study estimated the cost-effectiveness of prucalopride in the Dutch healthcare setting in 2011. The reason for choosing this date was that this was when clinical data were generated in the pivotal trials of prucalopride. Inflating costs to 2014 values did not significantly change the ICER of prucalopride relative to continued laxative treatment.

## Conclusion

In terms of cost per QALY gained, prucalopride was cost-effective in Dutch patients with chronic constipation that was inadequately relieved by the use of laxatives, as shown by a conservative base-case analysis using a Markov model. Extensive sensitivity analyses and scenario analyses underlined the robustness of the base-case cost-effectiveness.

## Author contributions

MN and DD developed the cost-effectiveness model and drafted the manuscript. AJ and LA provided guidance on data interpretation and revised the manuscript critically for important content. All authors provided final approval of the manuscript.

### Conflict of interest statement

M. Nuijten and D. Dubois are consultants to Shire; A. Joseph is an employee and stockholder of Shire; L. Annemans has acted as an advisor to Shire-Movetis.

## Footnotes

^1^Prucalopride Summary of Product Characteristics. Available online at: http://www.ema.europa.eu/docs/en_GB/document_library/EPAR_-_Product_Information/human/001012/WC500053998.pdf (Accessed March 2015)

^2^http://nhg.artsennet.nl/kenniscentrum/k_richtlijnen/k_nhgstandaarden/Samenvattingskaartje-NHGStandaard/M94_svk.htm

^3^CHMP ASSESSMENT REPORT FOR Resolor. www.ema.europa.eu/docs/en_GB/document_library/EPAR_-_Public_assessment_report/human/001012/WC500053997.pdf

^4^https://www.farmacotherapeutischkompas.nl/

## References

[B1] Annells M. Koch T. (2003). Constipation and the preached trio: diet, fluid intake, exercise. Int. J. Nurs. Stud. 40, 843–852. 10.1016/S0020-7489(03)00075-0 14568365

[B2] Briggs A. H. (2000). Handling uncertainty in cost-effectiveness models. Pharmacoeconomics 17, 479–500. 10.2165/00019053-200017050-00006 10977389

[B3] Briggs A. H. Goeree R. Blackhouse G. O'Brien B. J. (2002). Probabilistic analysis of cost-effectiveness models: choosing between treatment strategies for gastroesophageal reflux disease. Med. Decis. Making 22, 290–308. 10.1177/027298902400448867 12150595

[B4] Camilleri M. Kerstens R. Rykx A. Vandeplassche L. (2008). A placebo-controlled trial of prucalopride for severe chronic constipation. N. Engl. J. Med. 358, 2344–2354. 10.1056/NEJMoa0800670 18509121

[B5] Camilleri M. Van Outryve M. J. Beyens G. Kerstens R. Robinson P. Vandeplassche L. (2010). Clinical trial: the efficacy of open-label prucalopride treatment in patients with chronic constipation – follow-up of patients from the pivotal studies. Aliment. Pharmacol. Ther. 32, 1113–1123. 10.1111/j.1365-2036.2010.04455.x 21039673

[B6] Christiansen J. Rasmussen O. O. (1996). Colectomy for severe slow-transit constipation in strictly selected patients. Scand. J. Gastroenterol. 31, 770–773. 10.3109/00365529609010350 8858745

[B7] Chung B. D. Parekh U. Sellin J. H. (1999). Effect of increased fluid intake on stool output in normal healthy volunteers. J. Clin. Gastroenterol. 28, 29–32. 10.1097/00004836-199901000-00006 9916661

[B8] Dennison C. Prasad M. Lloyd A. Bhattacharyya S. K. Dhawan R. Coyne K. (2005). The health-related quality of life and economic burden of constipation. Pharmacoeconomics 23, 461–476. 10.2165/00019053-200523050-00006 15896098

[B9] Diemel J. M. Van Den Hurk A. P. J. M. Muris J. W. M. (2010). NHG-standaard obstipatie. Hisarts Wet. 53, 484–498 10.1007/BF03089256

[B10] Dubois D. Gilet H. Viala-Danten M. Tack J. (2010). Psychometric performance and clinical meaningfulness of the Patient Assessment of Constipation-Quality of Life questionnaire in prucalopride (RESOLOR) trials for chronic constipation. Neurogastroenterol. Motil. 22, e54–e63. 10.1111/j.1365-2982.2009.01408.x 19761492

[B11] Dukas L. Willett W. C. Giovannucci E. L. (2003). Association between physical activity, fiber intake, and other lifestyle variables and constipation in a study of women. Am. J. Gastroenterol. 98, 1790–1796. 10.1111/j.1572-0241.2003.07591.x 12907334

[B12] Duncan A. Forrest J. A. (2001). Surreptitious abuse of magnesium laxatives as a cause of chronic diarrhoea. Eur. J. Gastroenterol. Hepatol. 13, 599–601. 10.1097/00042737-200105000-00023 11396544

[B13] Duncan A. Morris A. J. Cameron A. Stewart M. J. Brydon W. G. Russell R. I. (1992). Laxative induced diarrhoea–a neglected diagnosis. J. R. Soc. Med. 85, 203–205. 1433059 10.1177/014107689208500408 PMC1294723

[B14] Frank L. Kleinman L. Farup C. Taylor L. Miner P. Jr. (1999). Psychometric validation of a constipation symptom assessment questionnaire. Scand. J. Gastroenterol. 34, 870–877. 10.1080/003655299750025327 10522604

[B15] Galligan J. J. Vanner S. (2005). Basic and clinical pharmacology of new motility promoting agents. Neurogastroenterol. Motil. 17, 643–653. 10.1111/j.1365-2982.2005.00675.x 16185302

[B16] Handleiding voor kostenonderzoek. (2010). Methoden en Standaard Kostprijzen Voor Economische Evaluaties in de Gezondheidszorg [Manual for Costing: Methods and Standard Costs for Economic Evaluations in Health Care]. Diemen: Handleiding voor Kostenonderzoek; College voor Zorgverzekeringen.

[B17] Johanson J. F. Kralstein J. (2007). Chronic constipation: a survey of the patient perspective. Aliment. Pharmacol. Ther. 25, 599–608. 10.1111/j.1365-2036.2006.03238.x 17305761

[B18] Jones M. P. Talley N. J. Nuyts G. Dubois D. (2002). Lack of objective evidence of efficacy of laxatives in chronic constipation. Dig. Dis. Sci. 47, 2222–2230. 10.1023/A:1020131126397 12395895

[B19] Ke M. Zou D. Yuan Y. Li Y. Lin L. Hao J.. (2012). Prucalopride in the treatment of chronic constipation in patients from the Asia-Pacific region: a randomized, double-blind, placebo-controlled study. Neurogastroenterol. Motil. 24, 999–e541. 10.1111/j.1365-2982.2012.01983.x 22882724 PMC3509366

[B20] Longstreth G. F. Thompson W. G. Chey W. D. Houghton L. A. Mearin F. Spiller R. C. (2006). Functional bowel disorders. Gastroenterology 130, 1480–1491. 10.1053/j.gastro.2005.11.061 16678561

[B21] Marquis P. De La Loge C. Dubois D. McDermott A. Chassany O. (2005). Development and validation of the Patient Assessment of Constipation Quality of Life questionnaire. Scand. J. Gastroenterol. 40, 540–551. 10.1080/00365520510012208 16036506

[B22] Meshkinpour H. Selod S. Movahedi H. Nami N. James N. Wilson A. (1998). Effects of regular exercise in management of chronic idiopathic constipation. Dig. Dis. Sci. 43, 2379–2383. 10.1023/A:1026609610466 9824122

[B23] Müller-Lissner S. Rykx A. Kerstens R. Vandeplassche L. (2010). A double-blind, placebo-controlled study of prucalopride in elderly patients with chronic constipation. Neurogastroenterol. Motil. 22, 991–998. 10.1111/j.1365-2982.2010.01533.x 20529205

[B25] NICE technology appraisals TA211. (2010). Prucalopride for the Treatment of Chronic Constipation in Women. Available online at: http://publications.nice.org.uk/prucalopride-for-the-treatment-of-chronic-constipation-in-women-ta211/guidance (Accessed December 2010)

[B26] Parker M. Haycox A. Graves J. (2011). Estimating the relationship between preference-based generic utility instruments and disease-specific quality-of-life measures in severe chronic constipation: challenges in practice. Pharmacoeconomics 29, 719–730. 10.2165/11588360-000000000-00000 21635017

[B27] Passmore A. P. Wilson-Davies K. Stoker C. Scott M. E. (1993). Chronic constipation in long stay elderly patients: a comparison of lactulose and a senna-fibre combination. BMJ 307, 769–771. 10.1136/bmj.307.6907.769 8219947 PMC1696423

[B28] Pikarsky A. J. Singh J. J. Weiss E. G. Nogueras J. J. Wexner S. D. (2001). Long-term follow-up of patients undergoing colectomy for colonic inertia. Dis. Colon Rectum 44, 179–183. 10.1007/BF02234290 11227933

[B29] Quigley E. M. Vandeplassche L. Kerstens R. Ausma J. (2009). Clinical trial: the efficacy, impact on quality of life, and safety and tolerability of prucalopride in severe chronic constipation – a 12-week, randomized, double-blind, placebo-controlled study. Aliment. Pharmacol. Ther. 29, 315–328. 10.1111/j.1365-2036.2008.03884.x 19035970

[B30] Read N. W. Celik A. F. Katsinelos P. (1995). Constipation and incontinence in the elderly. J. Clin. Gastroenterol. 20, 61–70. 10.1097/00004836-199501000-00016 7884183

[B31] Richtlijnen voor farmaco-economisch onderzoek. (2006). Richtlijnen voor Farmaco-Economisch Onderzoek. Diemen: Geactualiseerde versie. College voor Zorgverzekeringen.

[B32] Romero Y. Evans J. M. Fleming K. C. Phillips S. F. (1996). Constipation and fecal incontinence in the elderly population. Mayo Clin. Proc. 71, 81–92. 10.4065/71.1.81 8538239

[B33] Schiller L. R. (2004). New and emerging treatment options for chronic constipation. Rev. Gastroenterol. Disord. 4(Suppl. 2), S43–S51. 15184816

[B34] Singh G. Kahler K. Bharathi V. (2005). Constipation in adults: complications and comorbidities. Gastroenterology 128(Suppl. 2), A-154.

[B35] Singh G. Lingala V. Wang H. Vadhavkar S. Kahler K. H. Mithal A.. (2007). Use of health care resources and cost of care for adults with constipation. Clin. Gastroenterol. Hepatol. 5, 1053–1058. 10.1016/j.cgh.2007.04.019 17625982

[B36] Tack J. Quigley E. Camilleri M. Vandeplassche L. Kerstens R. (2013). Efficacy and safety of oral prucalopride in women with chronic constipation in whom laxatives have failed: an integrated analysis. United Eur. Gastroenterol. J. 1, 48–59. 10.1177/2050640612474651 24917940 PMC4040783

[B37] Tack J. Stanghellini V. Dubois D. Joseph A. Vandeplassche L. Kerstens R. (2014). Effect of prucalopride on symptoms of chronic constipation. Neurogastroenterol. Motil. 26, 21–27. 10.1111/nmo.12217 24106924 PMC4282451

[B38] Tack J. van Outryve M. Beyens G. Kerstens R. Vandeplassche L. (2009). Prucalopride (Resolor) in the treatment of severe chronic constipation in patients dissatisfied with laxatives. Gut 58, 357–365. 10.1136/gut.2008.162404 18987031

[B39] Taylor R. R. Guest J. F. (2010). The cost-effectiveness of macrogol 3350 compared to lactulose in the treatment of adults suffering from chronic constipation in the UK. Aliment. Pharmacol. Ther. 31, 302–312. 10.1111/j.1365-2036.2009.04191.x 19886948

[B40] Wald A. Scarpignato C. Kamm M. A. Mueller-Lissner S. Helfrich I. Schuijt C.. (2007). The burden of constipation on quality of life: results of a multinational survey. Aliment. Pharmacol. Ther. 26, 227–236. 10.1111/j.1365-2036.2007.03376.x 17593068

