# Supplementary material for: Cost-effectiveness of prucalopride in the treatment of chronic constipation in the Netherlands
Source: Front Pharmacol. 2015 Apr 14;6:67. doi: 10.3389/fphar.2015.00067 (PMC4396353; doi:10.3389/fphar.2015.00067)
Supplement: Supplementary file 1 [file DataSheet1.DOCX]

**Supplementary material**

**Scenario analyses**

The results of the scenario analyses are reported herein and the main findings are as follows:

- The ICER for prucalopride treatment relative to continued laxative treatment was € 9,015 per QALY gained in the base case analysis.
- The scenario analyses with highest impact on ICER were:
  - Excluding the stopping rule at 4 weeks: the ICER increased to € 16,665 per QALY gained.
  - Excluding cost of complications: the ICER increased to € 12,216 per QALY gained.
  - Using an average daily dose of 1.5 mg for prucalopride rather than the 2 mg daily dose used in the base-case analysis: the ICER decreased to € 4,974 per QALY gained.
- The scenario analyses also showed that:
- Including indirect costs due to lost productivity was a conservative approach, as the ICER decreased to € 5,228 per QALY gained.
- The time horizon of 1 year in base case analysis, was also conservative, as a time horizon of 3 years instead of 1 year decreased the ICER to € 4,436 per QALY gained.
- Inflating costs to 2014 values had minimal impact on the ICER.

**Table S1. Results of scenario analyses indicating the costs, QALYs, and ICER associated with prucalopride vs. continued laxative treatment for chronic constipation (DBC, Dutch diagnosis treatment code; ICER, incremental cost-effectiveness ratio; PAC-QoL, Patient Assessment of Constipation Quality of Life; QALY, quality-adjusted life-year; SCBM, spontaneous complete bowel movement).**

|  | **Costs (**€) | **QALYs** | **ICER (**€) |
| --- | --- | --- | --- |
| **Women: ≥ 3 SCBMs per week (base case: men and women ≥ 3 SCBMs per week)** |  |  |  |
| Prucalopride | 2,509 | 0.834 |  |
| Continued laxative treatment | 2,449 | 0.826 |  |
| Difference | 60 | 0.008 | 7,773 |
| **Satisfaction subscale PAC-QoL – total population (at least 1 point improvement on scale varying from 0 to 4 (base case: ≥ 3 SCBMs per week)** |  |  |  |
| Prucalopride | 2,435 | 0.843 |  |
| Continued laxative treatment | 2,369 | 0.830 |  |
| Difference | 66 | 0.013 | 5,197 |
| **No stopping rule (base case: include stopping rule)** |  |  |  |
| Prucalopride | 2,651 | 0.830 |  |
| Continued laxative treatment | 2,503 | 0.821 |  |
| Difference | 148 | 0.009 | 16,665 |
| **Exclude complications (base case: include complications)** |  |  |  |
| Prucalopride | 2,355 | 0.833 |  |
| Continued laxative treatment | 2,267 | 0.826 |  |
| Difference | 88 | 0.007 | 12,216 |
| **Dosing 1.5 mg (50% 1 mg; 50% 2 mg)**  **(base case: 2 mg)** |  |  |  |
| Prucalopride | 2,482 | 0.833 |  |
| Continued laxative treatment | 2,446 | 0.826 |  |
| Difference | 36 | 0.007 | 4,974 |
| **Include indirect costs (base case: exclude indirect costs)** |  |  |  |
| Prucalopride | 2,695 | 0.833 |  |
| Continued laxative treatment | 2,657 | 0.826 |  |
| Difference | 38 | 0.007 | 5,228 |
| **Exclude transportation costs (base case: include transportation costs)** |  |  |  |
| Prucalopride | 2,420 | 0.833 |  |
| Continued laxative treatment | 2,353 | 0.826 |  |
| Difference | 67 | 0.007 | 9,244 |
| **Hospital costs based on daily cost (base case: costs based on DBC)** |  |  |  |
| Prucalopride | 2,405 | 0.833 |  |
| Continued laxative treatment | 2,323 | 0.826 |  |
| Difference | 82 | 0.007 | 11,399 |
| **Time horizon: 3 years (base case: 1 year)** |  |  |  |
| Prucalopride | 6,557 | 2.48 |  |
| Continued laxative treatment | 6,443 | 2.46 |  |
| Difference | 114 | 0.03 | 4,436 |
| **Mortality (base case: no mortality)** |  |  |  |
| Prucalopride | 2,507 | 0.832 |  |
| Continued laxative treatment | 2,442 | 0.825 |  |
| Difference | 66 | 0.007 | 8,883 |
| **Inflating costs to 2014 (2.5% in 2012 and 2013),** |  |  |  |
| Prucalopride | 2,638 | 0.833 |  |
| Continued laxative treatment | 2,570 | 0.826 |  |
| Difference | 68 | 0.007 | 9,714 |
|  | | | |
